# Reducing treatment delay for early intervention: evaluation of a community based crisis helpline

**DOI:** 10.1186/1744-859X-11-20

**Published:** 2012-07-24

**Authors:** Amresh K Shrivastava, Megan E Johnston, Larry Stitt, Meghana Thakar, Gopa Sakel, Sunita Iyer, Nilesh Shah, Yves Bureau

**Affiliations:** 1 Department of Psychiatry, Silver Mind Hospital and Mental Health Foundation of India, (PRERANA Charitable Trust) Mumbai, University of Western Ontario, London, Canada; 2 Department of Psychology, University of Toronto, Toronto, ON, Canada; 3 Department of Epidemiology & Biostatistics, Biostatistical Support Unit, The University of Western Ontario, London, Ontario, Canada; 4 Silver mind Hospital, Mumbai, Child and Adolescent Mental Health Practitioner, Lambeth CAMHS Early Intervention Team, South London and Maudsley NHS Trust, London, UK; 5 PRERANA, suicide prevention services, Mental Health Foundation of India, (PRERANA Charitable Trust), Mumbai, India; 6 PRERANA, suicide prevention services, Mental Health Foundation of India, (PRERANA Charitable Trust), Mumbai, India; 7 LTMG hospital, University of Mumbai, Mumbai, India; 8 University of Western Ontario, 1151 Richmond Street, London, Ontario, N6A 3 K7, Canada; 9 Research Scientist Lawson Health Research Institute, 268 Grosvenor Street, London, Ontario, N6A 4 V2, Canada; 10 Regional Mental Health Care, 467, Sunset Drive, St.Thomas, Ontario, N5H 3 V9, Canada

**Keywords:** Helpline, Stigma, Psychosis, Early intervention, Duration of treatment

## Abstract

**Background:**

A limited number of studies have assessed the pathways to care of patients experiencing psychosis for the first time. Helpline/clinic programs may offer patients who are still functional but have potential for crisis an alternative that is free from judgment.

**Methods:**

In this study we report on patient calling a round-the-clock crisis helpline for suicide prevention supported by psychiatric facilities in Mumbai, India. Chi-square and test of mean differences were used to compare outcomes between first-episode patients and those with a previous history.

**Results:**

Within five years, the helpline received 15,169 calls. Of those callers, 2341 (15.4%) experienced suicidal ideation. Two hundred and thirty four patients opting for counseling lasting 12 months agreed to a psychiatric assessment. Of those, 32 were fist time psychosis sufferers, whereas, 54 had previously been psychotic. Of all psychiatric assessments, the clinic received 94 patients with ‘first-episode psychosis’. We found that the duration of illness was significantly shorter (17 vs. 28 months) and suicide attempts were fewer (16 vs. 21) in first-time psychosis sufferers compared to those with a treatment history.

**Conclusions:**

We conclude that some first-episode patients of schizophrenia and other disorders do access services by using helplines. We also argue that helplines may be somewhat immune to stigma, allowing patients a safe alternative when finding help.

## Introduction

There is little doubt that patients experiencing psychosis benefit greatly from early intervention. Consequently, the mental health community has concentrated on bringing mental health services to patients as quickly as possible. In order to facilitate contact with mental health professionals (MHP), mental health centers are implementing early intervention programs with innovative strategies such as helpline services and clinic access. It is curious; however, that most studies investigating mental health care access have focused on hospital-based psychiatric interventions while little attention has been spent investigating the use of helpline programs. This is perhaps not surprising as traditionally, individuals presenting with psychosis are likely to be taken to the hospital for treatment in favor of other facilities. Secondly, there is still great stigma associated with mental health problems, leading individuals and family members to avoid seeking treatment until absolutely necessary [1]. Individuals presenting with psychosis are especially afraid of being diagnosed as schizophrenic, not realizing that there are a number of causes for psychosis. Patients, then, if not opting for a hospital may go to clinics not advertising mental health services, perhaps to hide their primary reason for seeking help.

Duration of untreated psychosis (DUP) is an important prognostic variable [2,3]. Early detection programs are required to decrease the period between illness onset, diagnosis and treatment in first-episode psychotic patients. Long duration of illness is associated with poor outcome in schizophrenia [4]. It is reasonable then that early treatment should prevent psychosis or limit the damage caused by psychosis which is supported by neurobiological, and phenomenological data [5]. Studies have also shown that determinants of DUP that facilitate early treatment are education, awareness, and research to name a few. It is imperative that any strategy implemented for early intervention be culturally sensitive and pragmatic. What works in North American and European countries may not necessarily work in India due to cultural perceptions of mental illness [6]. Consequently, people of different cultures may choose to seek help at later or earlier stages of an illness or may choose alternatives not considered by others.

A study by Bechard-Evans et al. [7] showed that longer DUP (help seeking component) was significantly associated with earlier age of onset, diagnosis of schizophrenia spectrum psychosis, and poor pre-morbid adjustment during adolescence. Longer DUP (referral component) was associated with earlier age of onset and first help-seeking contact having been made with a non-medical professional [7]. Although there is agreement with respect to an association between delay in treatment of psychosis and outcome, little is known regarding how patients suffering from a first psychotic episode find help. The process of finding help is complex and involves a diverse range of contacts. It is also likely to influence treatment delay [8]. This was explored in the present research.

A limited number of studies have assessed the pathways to care of patients experiencing psychosis for the first time. A recent study by Cougnard et al. [9] examined how patients of psychosis obtained care from the onset to being admitted. Twelve percent of subjects were first admitted without any previous-contact with a helping individual (MHP, general practitioners, others…). For approximately 70% of patients, the first helping contact was a health care professional. Thus, delay in access to care does not appear to result primarily from inadequate management by health care professionals, suggesting there may be reluctance from patients to seek help in the first place. Clearly, tools that allow for easy, stigma-free contact with MHP should enhance early intervention efforts [9].

As previously mentioned, hospital-based mental health facilities receive mental health patients who are already in crisis. Helpline/clinic programs may offer patients who are still functional but have potential for crisis an alternative that is free from judgment, allows for the complete description of symptoms, provides an active listener, and is free from financial cost. A person experiencing distress in all aspects of their lives can rely on such a service for suggestions relating to treatment or to facilitate a visit to a mental health professional. These aspects of helplines to our knowledge have not been sufficiently assessed.

In this study we report on a community-based clinic with a crisis helpline aimed at the prevention of suicide from the metropolitan city of Mumbai. We describe some demographics behind helpline use, focusing on psychotic and first-time psychotic sufferers calling the helpline. Of special interest was the prevalence of patients seeking help following the call.

## Method

A helpline was implemented for crisis and suicide intervention for the entire city of Mumbai. This was the first helpline in Mumbai for the prevention of suicide and for mental health crises in general which was publicly advertised by the media and in press conferences. It was based in a residential location in the community. This helpline was available 24 hour per day and seven day per week with trained mental health professionals receiving all calls. It is important to note that those manning the telephones were not volunteers. Specifically, they were trained psychologists, social workers, and therapist counselors. Callers were provided with two options when they called. They were given the option of “counseling” or “psychiatric assessment.” Within the facility, psychiatric assessment was available, and patients were not obligated to visit a hospital. Some patients came to the centre and opted for counseling, some stayed with counselors for therapy, and others agreed to psychiatric assessments. Those who stayed with counselors for one year were also assessed by psychiatrists. Duration of illness and the general history of the patient were determined from questions to the patient and family and by the professional opinion of the psychiatrist.

### Data collection and statistics

All data were collected by mental health professionals at the time of the call and any following assessment. The database was compiled from the records and frequencies and tests of means analyzed using SAS (Statistical Analysis System inc, version 9.1, NC, USA, 2009). All data are reported as frequency, percentage, and means. When appropriate odds ratios (OR) with confidence intervals were calculated by standard formulations. Tests of association between nominal variables were conducted using the Chi-Square test. Means were compared using the independent samples *t*-test. All tests were considered significant when the Type 1 error was less than 5% (*p* < .05). Formal ethics were not required as this is an evaluation of a service. Consequently, patient permission is not required to publish this data.

## Results

Over a five year span, the helpline received 15,169 calls. Of those callers, 2,341 (15.4%) experienced suicidal ideation. These callers visited the outpatient clinic for assessment and subsequent psychosocial intervention. From the callers that experienced suicidal ideation, 781 (34%) were assessed by psychiatrists (Table 1), whereas, 1560 (66%) received psychosocial assessment and intervention. Of those receiving psychosocial assessment and intervention, 234 (15%) remained in therapy and at the end of 12 months consented to psychiatric assessment (Table 2). Of the 234 patients who remained in counseling 94 (40%) were observe to have early psychosis. Thirty two (17.7%) of the 94 patients were seen by a psychiatrist following 12 months of counseling, whereas 62 (30.5%) were seen by a psychiatrist shortly after the call to the hotline (Table 3). Of the 94 psychosis sufferers, 54 (57.4%) were previous-contact patients, with 22 (40.7%) of the 54 having a non-affective psychosis (Table 2).

**Table 1:** Frequency and percentage for diagnoses by treatment history

**Diagnosis**	**Psychiatric assessment N = 781**					
	**Patients assessed on clinic Group 1**	**Psychiatric treatment history**	**First-contact**	**OR**	**95% CI**	**P-value**
All diagnoses	781 [24%]	578 [74%]	203 [26%][26%]			
Nonaffective psychosis	187 [23.9]	122	62	0.608	0.445 – 0.872	<.01
MMD	83 [10.6]	51	32	0.517	0.322 – 0.831	<.01
SUD	78 [9.9]	67	11	2.288	1.184 – 4.422	<.05
Anxiety-depression	178 [22.7]	120	58	0.655	0.454 – 0.943	<.05
Bipolar disorder	22 [2.8]	14	8	0.605	0.250 – 1.464	n.s.
PD	85 [10.8]	83	2	16.85	4.106 – 69.167	<0.001
No Diagnosis	151 [16.1]	121	30	1.527	0.987 – 2.362	n.s.

Frequency and percentage for diagnoses by treatment history. Odd ratios (OR) are shown indicating the relative representation of diagnoses by treatment history. A result was considered significant when the probability of a Type 1 error was less than 5% (*p* < .05).

n.s. = not significant.

MMD = Major Mood Disorder, SUD = Substance Use Disorder, PB = Personality Disorder.

**Table 2:** Frequency and percentage for diagnoses treatment history for those patients assessed by a psychiatrist after 12 months of counseling

**Diagnosis**	**Psychiatric assessment after 12 months of counseling**					
	**Patients assessed on clinic (N = 234) Groups 2**	**Psychiatric treatment history N = 54**	**First-contact N = 180**	**OR**	**95% CI**	**P-value**
	234	54 [23%]	180 [77%]			
Nonaffective psychosis	54	22	32	3.18	1.637 –6.176	>.01
MMD	70	11	59	0.525	0.252 –1.091	n.s.
SUD	28	6	22	0.9	0.344 –2.342	n.s.
Anxiety-depression	43	12	31	1.373	0.649 –2.905	n.s.
Bipolar disorder	11	3	8	1.265	0.324 –4.944	n.s.
PD	2	00	2	0	0–NaN	N/A
No Diagnosis	26	00	26	0	0–NaN	N/A

Frequency and percentage for diagnoses treatment history for those patients assessed by a psychiatrist after 12 months of counseling. Odd ratios (OR) are shown indicating the relative representation of diagnoses by treatment history. A result was considered significant when the probability of a Type 1 error was less than 5% (*p* < .05).

n.s. = not significant, NaN = not a number.

MMD = Major Mood Disorder, SUD = Substance Use Disorder, PB = Personality Disorder.

**Table 3:** Frequency distribution and percent of first-contact and previous-contact patients categorized by direct assessment (Gr1) or assessment following 12 months of counseling (Gr2) by diagnosis

	**First-contact**			**Previous-contact**			**Total**	**Statistical**
	**Gr1**	**GR 2**	**Gr 1 + 2**	**Gr1**	**Gr2**	**Gr 1 + 2**			
	**203 [20%]**	**180 [18%]**	**383 [38%]**	**578 [57%]**	**54 [5%]**	**632 [62%]**	**1015**		
Psychosis	62	32	94	122	22	144	248	χ ^2^(1) = 11.4, *p* < .01
MMD	32	59	91	51	11	62	153	χ ^2^(1) = 33.0, *p* <0.001
SUD	11	22	33	67	6	73	106	χ ^2^(1) = 39.9, *p* < .001
Anxiety-depression	58	31	89	120	12	132	221	χ ^2^(1) = 22.5, *p* <0.001
Bipolar	8	8	16	14	3	17	33	χ ^2^(1) = 3.88, *p* < .05
PD	2	2	4	83	0	83	87	χ ^2^(1) = 42.5, *p* < .001
No diagnosis	30	26	56	121	0	121	177	χ ^2^(1) = 65.9, *p* < .001

Frequency distribution and percent of first-contact and previous-contact patients categorized by direct assessment (Gr1) or assessment following 12 months of counseling (Gr2) by diagnosis. Percent of total indicated. Chi-Square analyses were conducted to determine the association between groups and patient history separately for each diagnosis. A result was considered significant when the probability of a Type 1 error was less than 5% (*p* < .05).

n.s. = not significant.

MMD = Major Mood Disorder, SUD = Substance Use Disorder, PB = Personality Disorder.

Overall, 15% of patients using the helpline accessed the care from a mental health professional or psychiatrist. Of these patients, 1015 (43.3%) could be fully assessed for diagnosis. Thus, 94 (9.2%) were never treated, whereas, 144 (14.1%) were previous-contact patients of early phase psychosis. Of the 1015 patients with suicidal ideation assessed in the community clinic, 383 (37.7%) were first-contact patients. Of the group diagnosed with non-affective psychosis, 94 (39.5%) patients were first-contact (early psychosis) and 144 (60.5%) had a history of treatment for schizophrenia (Table 3). The duration of illness in the first-contact psychosis group was significantly lower (17 months) in comparison to patients with previous psychiatric contact (53 months), *t* (236) = 48.247, *p* < .001. In the first-contact group, 16 (17%) had a history of suicide attempts compared to 21 (14.5%) in the previous psychiatric contact patients. This association was not significant, *χ*^2^(1) = 0.257, *p* = n.s.

A range of psychiatric diagnoses were seen in the first-contact group that had never been treated, such as major mood disorders (MMD), anxiety-depression, substance use disordered (SUD), and personality disorders (PD). Fifty-six (14.6%) patients in the group that had never been treated did not qualify for any axis 1 or axis II DSM IV diagnosis (Table 3).

In group 1 (psychiatric assessment, Table 1), the results indicate that patients with bipolar disorder were represented equally among patients with a history of psychiatric treatment and first-contact patients. SUD patients had a greater representation in the treatment history group compared to the first-contact group (OR = 2.288, *p* < .05). For all other diagnoses, there was a greater representation in the first-contact patients (OR range = 0.517 to 2.289, *p*’s range < .05 to < .01; see Table 1). In group II (psychosocial assessment and intervention group, Table 2) the results suggest that there were no differences in representation between first-contact and previous-contact patients in diagnoses of MMD, SUD, anxiety-depression, bipolar disorder, PD (Table 2). For nonaffective psychosis there was a greater representation in the treatment history group, OR = 3.18, *p* < .01, whereas, there was a greater representation of no diagnosis for the first-contact group (Table 2).

Finally, when comparing first-contact and previous-contact patients in the two assessment groups, the results show that for patients with all diagnoses, those who received direct psychiatric assessment and those who received psychiatric assessment from group counseling differed significantly in whether they were first-contact patients or were patients who had previously been in contact with mental health professionals. First-contact patients of all diagnoses were less likely to receive direct psychiatric assessment than patients with previous-contact. On the other hand, first-contact patients with all diagnoses were more likely to receive psychiatric assessment from group counseling than patients who had previous-contact (*χ*^2^ range = 11.4 to 65.9, *p*’s range = <.01 to < .05; see Table 3).

## Discussion

Helpline services are a welcome addition to an ever growing toolset for tending to mental health issues. It is especially important to offer an opportunity for early contact, identification, and treatment for mental disorders as well as to those with psychosocial problems. When the crisis callers attend an outpatient clinic the advantages increase in terms of accessibility for individuals with previously untreated illnesses, particularly early psychoses.

### Access to care

Several strategies have been adopted to reach out to patients who are in the early phase of illness with education being the best [10]. General practitioners, public education, and school programs have made use of the internet, printed promotional material, lectures, and seminars. Workshops have also been used by early intervention programs in order to reduce stigma and raise confidence that using the programs will be beneficial.

In Indian culture, particularly in Mumbai where this study was conducted, there is an open-door system. A patient does not need to be referred by an agency to attend a walk-in centre. Patients in this culture have a range of choices available for obtaining help and treatment. They approach faith healers, religious leaders, physicians of alternative medicine, family physicians, psychiatrists, counselors, psychiatric social workers, volunteers of social groups, voluntary agencies, and various support groups. A crisis helpline is only one of many options available to patients and families for obtaining information, opportunities for discussion, interventions, and even referrals.

In spite of an open door policy, families generally seek professional help only when symptom severity escalates to the point where there is danger to the patient or care-givers. This reluctance to seek help is likely due to stigma; the fear of being labeled with a mental illness results in a resistance to seek treatment. A limited number of studies have assessed the pathways to care of patients experiencing psychosis for the first time. In the present study, patients received access to care using a helpline. Sixty six percent of patients decided to see a mental health professionals, whereas, only 33% chose to see a psychiatrist. Furthermore, of all patients who were first-contact psychotics (94), 32 (17%) of patients were assessed immediately by a psychiatrist, whereas, 62 (30.5%) were assessed after 12 months of counseling. It is clear that in the early phase of illness patients and relatives prefer to see a mental health professional other than a psychiatrist. This trend may be related to stigma as a patient may defer being diagnosed for as long as possible with the hope that counseling will be sufficient. We found that a professional is most likely to be contacted by telephone when the situation is urgent. A similar trend is observed for patients with past and ongoing treatment history. This study suggests that early phase patients prefer to contact non-psychiatric mental health professions. Crisis helplines also provide ready access to early psychosis patients who have never been treated; this can help prevent a delay in treatment. Health service development efforts in early intervention need to consider establishing non-hospital based community services integrated with help lines to expand the network for early identification.

In our study we observed that diagnoses were not equally distributed among patients with or without treatment history. First-contact patients were more likely to be in psychosis, be MMD, or anxious-depressed, but less likely to be SUD, or PD compared to patients with a treatment history. In patients that received counseling for 12 months the pattern was somewhat different. First-contact patients were less likely to be psychotic but more likely to be without diagnosis then those patients with a treatment history. This suggests that first-contact patients are more likely to be in crisis and have chosen to access the helpline. Perhaps the anonymity conferred by the helpline is conducive to seeking help and obtaining advice from a MHP from which a reasonable course of action can be taken that is free from judgment.

When considering patients who where directly assessed after calling the helpline and those assessed after 12 months of counseling, it was evident that there were relatively more patients diagnosed in the counseling group compared to the direct assessment group. This was true for all categories. Patients that required counseling for 12 months were possibly more troubled than those agreeing to an immediate assessment. We have no data to suggest a mechanism. However, there may have been social/cultural reasons preventing an immediate diagnosis. Stigma, as mentioned, may indeed be the major reason for this phenomenon.

### Treatment delay, duration of untreated psychosis, and never-treated patients

DUP is an important prognostic variable [2,3]. It is known that long illness duration is associated with poor outcome in schizophrenia. In our study we show that duration of illness is significantly shorter in the ‘first-contact’ group (17 months) compared to patients with a history of psychosis (26 months). It is possible then that helpline services can get patients help early in the illness process, preventing treatment delays. A caveat, however, is whether or not a first-contact person on subsequent calls will have an increase in DUP compared to their first-contact. Nevertheless, there are psychosocial and cultural factors influencing DUP and consequently on the treatment which is reported from low- and middle-income countries [11-14].

Although there is agreement on the association between delay in treatment of psychosis and outcome, less is known regarding how patients suffering a psychotic episode for the first time find help. In the present study we found that for both first-episode (n = 94) and previous-contact patients (n = 144) who had equal access to care, mean durations of illness were 17 and 53 months respectively. It is clear that the availability of crisis helplines can effectively reduce treatment delay and provide early and easy access for diagnosis and treatment. Some studies have argued that family members’ levels of knowledge of schizophrenia may not necessarily have a major impact upon the length of treatment delays. Early psychotic symptoms are often attributed to depression, lack of motivation or relational stressors. Family members’ decisions to seek help often were solidified only after the emergence of unbearable psychotic symptoms or socially disruptive behaviors. This is one explanation of why they are more likely to contact during times of crisis [15,16].

Individuals with a first-episode of psychotic illness are known to be at a high risk of suicide, yet little is understood about the timing of risk in this critical period of illness [12,13]. Another interesting finding is the rate of suicide attempts prior to contacting the hotline. Seventeen percent of never treated patients and 14.5% of patients with previous treatment had a history of suicide. The fact that the suicide rate is high amongst first-episode patients before contacting services is well known [17]. Contact through our crisis helpline demonstrated in this study offers an excellent opportunity for prevention by intervening early during illness. It has been consistently demonstrated that suicide attempts can be reduced during the first episode by treating them in early intervention programs. Helplines and community-located clinics can facilitate this initiative and should be encouraged.

## Conclusions

In this study we reported on a community-based clinic with a crisis helpline for the prevention of suicide from the metropolitan city of Mumbai. This clinic was not promoted for detection and treatment of early psychosis. However, in spite of this fact, we did make contact with a number of patients with psychotic disorder for whom we were able to help. We concluded that some first-episode patients of schizophrenia and other disorders do access the services by using helplines. However, more research is required to determine whether community-based round-the-clock helplines can be a supportive service to traditional-based services. The present study highlights four main points which can be useful in early intervention initiatives: 1. It is not necessary to popularize the term ‘psychosis’ to get the patients into treatment; 2. Centers based in the community where people live offer more comforting access to care; 3. People do recognize mental health issues and approach the easily available services; 4. Barriers in access to care in contrary to the very philosophy of ‘early intervention.’ If the concept has to be translated in reality, patients need to be seen whenever they need and wherever they live.

## Abbreviations

MHP, Mental health professionals; DUP, Duration of untreated psychosis; OR, Odds ratios; MMD, Major mood disorders; SUD, Substance use disordered; PD, Personality disorders.

## Competing interests

The authors declare that they have no competing interests.

## Authors’ contributions

AS conceptualized, designed, supervised and did psychiatric assessment for the study as principal investigator; MJ, was involved in interpretation of data and writing the manuscript; LS performed the statistical analysis; MT, GS, and SI did patient assessment, data management, and managed the helpline; NS supervised data management and psychiatric assessment; and YB undertook the responsibility of writing and finalizing the draft for submission. All authors read and approved the final manuscript.
